# Development and implementation of a municipal outdoor play policy for children and youth in Nova Scotia, Canada: a community case study

**DOI:** 10.3389/fpubh.2024.1334767

**Published:** 2024-03-06

**Authors:** Hilary A. T. Caldwell, Mike Arthur, Ashley Simms, Hannah Mawhinney, Camille L. Hancock Friesen, Sara F. L. Kirk

**Affiliations:** ^1^Healthy Populations Institute, Dalhousie University, Halifax, NS, Canada; ^2^Town of Truro, Truro, NS, Canada; ^3^Children’s Nebraska and University of Nebraska Medical Center, Omaha, NE, United States; ^4^School of Health and Human Performance, Dalhousie University, Halifax, NS, Canada

**Keywords:** adolescents, physical activity, healthy environments, outdoor play, urban, town, policy

## Abstract

**Background:** Children and youth benefit from outdoor play; however, environments and policies to support outdoor play are often limited. The purpose of this paper is to describe a case study of the development of a municipal outdoor play policy in Nova Scotia, Canada. The outdoor play policy was developed by the Town of Truro with support from the UpLift Partnership, a School-Community-University Partnership in Nova Scotia, Canada. UpLift supports the health and well-being of school-aged children and youth using a Health Promoting Schools approach which identifies the important role of municipal government in creating healthy school communities. The UpLift Partnership and the municipality hosted online workshops for municipal staff, community leaders and partners that included content about the importance of outdoor play, barriers and facilitators to outdoor play, best practices for youth engagement, the policy development process, and how policy actions can support outdoor play. Workshop participants developed policy actions for their community of Truro, Nova Scotia to increase opportunities for outdoor play for children and youth. Following the workshops, a small team from the municipality and UpLift drafted an outdoor play policy and submitted it to Truro town council for approval. The outdoor play policy was adopted in Fall 2021 and has since informed recreation and municipal planning decisions. By presenting a case study of the development of this outdoor play policy, we hope other communities may be inspired to develop and adopt their own outdoor play policies to benefit children and youth in their communities.

## 1 Introduction

Children and youth have the right to play and spend time in environments that promote a high-quality of life, as conceptualized by the Child Friendly City Framework (1). This Framework is committed to the implementation of the UN Convention on the Rights of the Child (2), and guarantees that every young person should be able to meet friends and play, walk safely in the streets on their own, participate in cultural and social events, and influence decisions about where they live, be it large or small, urban or rural (3). The Declaration on the Right to Play for Children in Canada calls on individuals and organizations to promote and protect the child’s right to play at local, regional and national levels (4). In 2019, building on the Child Friendly City Framework, the Canadian Public Health Association released its Play-Friendly City Framework (5). A Play-Friendly City considers children’s well-being and access to play in its design, involves children in decision-making, and prioritizes access to play in everyday environments (5). The Play-Friendly Cities Framework includes four criteria, each with individual actions, to improve the *playability* of a community: (1) participation of children in decision-making; (2) safe and active routes around the community; (3) safe and accessible informal play environments; and (4) evidence-informed design of formal play spaces (5). The Play-Friendly Cities Framework and Declaration on the Right to Play for Children in Canada are complementary—both prioritize child-led opportunities, and access and space for children to play in their communities (4, 5). When the Play-Friendly Cities Framework was used to guide a content analysis of Nova Scotian municipal physical activity and active transportation strategies, we revealed that actions related to safe and active routes around the community were most common and actions related to participation of children in decision making were least prevalent in the municipal strategies (6). All municipal strategies included some actions reflective of a Play-Friendly City, but there was great variability across municipalities, suggesting that further prioritization of opportunities for play is needed across the province (6).

Children and youth who regularly participate in recommended amounts of physical activity experience physical, socio-emotional, and cognitive benefits (7, 8). In children and youth, outdoor time is related to physical activity participation, such that physical activity is generally higher when children are outdoors versus when they are indoors (9). Outdoor time and outdoor play among children and youth are correlated with variables at the individual, parental, and community levels. At the community level, availability of recreation and physical activity facilities, play spaces and playgrounds, and rurality were positively associated with outdoor time and outdoor play (10). To increase child and youth physical activity levels, policies related to school environmental support (i.e., offer a variety of physical activities, enhance the built environment), community environmental support (i.e., interesting play spaces, community programs), and active transport/urban design may be most effective (11). Since the completion of our project in 2021, a child friendly neighborhood assessment index was developed that includes neighborhood and community features related to outdoor play for children and youth, including distances to parks, playgrounds and recreation amenities and number of bicycle lanes (12). During the COVID-19 pandemic, policies and public health guidelines initially restricted access to public recreation spaces to minimize gatherings, limiting Canadian children’s access to outdoor play (13, 14). As the pandemic progressed and Canadians were urged to spend more time outdoors, municipalities were essential in providing access to spaces for outdoor play. Policy and practice need to prioritize places and spaces for children and youth to be outdoors and physically active, particularly in their communities. The scope of places for children and youth to play must extend beyond formal playgrounds and parks to include a variety of public spaces that facilitate outdoor play.

The Position Statement on Active Outdoor Play suggests “increasing children’s opportunities for self-directed play outdoors in all settings—at home, at school, in childcare, the community and nature,” and recommends that governments collaborate across sectors to improve children’s access to outdoor play in nature and the outdoors (15). *Let’s Get Moving*, an action plan for increasing physical activity in Nova Scotia, has actions to encourage communities to develop policies that make it easier for Nova Scotians to incorporate less structured movement into their daily routines, and to create the conditions that support outdoor play and other active recreation choices (16). In recent years, policies related to play have been developed and implemented globally as a strategy to encourage mostly unstructured play in everyday spaces. The Canadian Public Health Association developed guidance on how to develop a play policy; however, specific details with examples of the development and implementation of municipal policies were not included. Municipal governments have a unique and necessary role in supporting outdoor play. They have legislated authority in key policy areas such as land use planning, active transportation, infrastructure, recreation programs, services, and facilities. Provincial legislation in Nova Scotia was changed in 2019 to require attention to recreation and parks in official municipal plans, which need to be approved by municipal governments (17). A play policy can assist municipalities in demonstrating their commitment to providing unstructured play opportunities, and such policies require collaborative decision-making that involves parents, children, child development experts, local leaders, local public health units, relevant government agencies (i.e., transportation, recreation), and local organizations related to play (i.e., family resource centres, youth-serving organizations) (5). A study of municipal officers in the United States identified that lack of political will, limited staff, and limited collaboration across municipal departments acted as barriers to the consideration of physical activity in community design and planning decisions (18). Despite these challenges, municipal play policies have been implemented in various communities globally, such as Calgary (Canada), Aberdeen (United Kingdom), and Palmerston North (New Zealand) (19–21).

For other communities to successfully develop their own play policies, it is essential to share experiences around the development and implementation of such policies. Community case studies can be used to document a local experience of delivering a service to meet the community’s needs, and include principles of community engagement to develop policies, programs, or services (22). Therefore, the purpose of this case study is to describe the development and implementation of outdoor play policies in Nova Scotia for children and youth and the outcomes and lessons learned in one community in Nova Scotia, Canada.

## 2 Context

### 2.1 UpLift partnership

The UpLift Partnership is a School-Community-University Partnership in Nova Scotia, Canada supporting the health and learning of school-aged children and youth using a Health Promoting Schools (HPS) approach (23, 24). The UpLift core team is hosted within Dalhousie University’s Healthy Populations Institute, while operating in partnership with the provincial government, Nova Scotia Health, school communities, non-profits, and the private sector. The work of the partnership is jointly funded by the Public Health Agency of Canada and the private sector to enhance HPS in six inter-related domains: (1) Partnerships and Leadership, (2) Planning and Evaluation, (3) Communication and Knowledge Exchange, (4) Capacity Building, (5) Child and Youth Engagement, and (6) School and Community Engagement and Action. HPS is a framework for schools to “constantly strengthen their capacity as a healthy setting for living, learning, and working” (25), and it is well recognized that HPS work requires partnerships from players outside schools, such as local or municipal governments (26). As part of the sixth domain (School and Community Engagement and Action), the UpLift Partnership engaged several communities in Nova Scotia through the Municipal Policy Project (MPP).

### 2.2 Municipal policy project

The goal of the MPP was to work directly with municipal recreation departments and leaders, physical activity leaders, and community partners to increase their knowledge, skills, and confidence in developing and implementing an outdoor play policy for their community. A virtual information session was hosted in Fall 2020 during which UpLift core team members introduced the MPP to Nova Scotia municipal recreation and physical activity staff, described the project and responsibilities of municipalities, and answered any questions. Following the virtual session, communities were asked to submit an Expression of Interest to participate in the project. In the Expressions of Interest, communities were asked to described: their level of commitment from management and/or elected officials, three major play activities for children/youth in their communities, key partners, and readiness for this project. At this time, we received four expressions of interest. One community withdrew from the project before the workshops due to staffing changes and capacity limitations.

## 3 Key programmatic elements

### 3.1 Municipal policy project workshops

The UpLift Partnership worked with the remaining three interested communities in 2020–2021 to support the development of municipal policy actions to promote outdoor play and active travel for children and youth. A small working group was formed in each community including 1–2 municipal staff and UpLift Partnership team members, as needed. The roles of municipal staff and the UpLift Partnership in the Municipal Policy Project are included in Table 1.

**Table 1:** Roles of municipal staff and the UpLift partnership in the municipal policy project.

UpLift core team role	Municipal staff role
Co-lead municipal working group Design and facilitate the virtual workshops Provide evidence-based resources on outdoor play Survey local high school students on outdoor play experiences and preferences Conduct research on municipal trends in outdoor play in Nova Scotia Write and revise the policy, based on feedback from municipal staff	Prepare and submit expressions of interest to UpLift Co-lead the working group Inventory existing outdoor play spaces, services and resources in the town Recruit community leaders and other municipal departments for virtual workshops Provide feedback on policy draft Advance policy for approval at municipal council Lead implementation of policy

The working group guided the design and implementation of the policy planning session, such as inviting external stakeholders, virtual workshop logistics, agenda and workshop design, and distribution of material to participants. Each community participated in a virtual workshop (two sessions x 2.25 h), hosted by the municipality and the UpLift Partnership and attended by invited community members. Workshop participants included representatives from municipal recreation, municipal planning departments, local colleges, public schools, provincial government departments such as education, health, recreation and community services, family resource centres, and community organizations/ non-profits (Table 2).

**Table 2:** Sample municipal policy project workshop agenda.

Time	Topic	Presenter/facilitator
Workshop 1		
9:45 AM	Welcome; review of workshop purpose and introductions	UpLift staff, municipal recreation staff, municipal leader (e.g., mayor)
10:05 AM	Outdoor play overview	UpLift staff
10:20 AM	Correlates of outdoor play and content analysis of Nova Scotia physical activity strategies	UpLift staff
10:35 AM	Community profile	Municipal recreation staff
10:50 AM	Data from youth input survey	UpLift youth engagement staff
11:20 AM	Barriers and enablers to outdoor play and active travel (small group activity)	UpLift staff
11:50 AM	Plan for next session	UpLift staff
Workshop 2		
9:45 AM	Municipal policy & policy development cycle	UpLift staff
10:00 AM	Share evidence and best practices with working group (each participant was assigned a reading assignment on outdoor play and then presented it during the session)	UpLift staff
10:45 AM	Create a municipal vision for outdoor play and active travel (in groups)	UpLift staff
10:55 AM	Brainstorm and report draft policy actions (in groups)	All participants
11:50 AM	Next steps and adjournment	UpLift staff

During the workshops, participants worked in small groups to identify potential outdoor play policy actions for their communities (see Table 3 for examples). Following the workshops, participants in each community were sent workshop proceedings, including the workshop slides and policy actions developed in the workshop.

**Table 3:** Examples of municipal policy project policy actions, as identified by workshop attendees.

Policy actions suggested by workshop attendees
The municipality will conduct an audit of existing playgrounds to ensure that they are challenging and connected to nature The municipality will conduct an inventory on rules and bylaws that inhibit outdoor play The municipality will provide public washroom access at all outdoor recreation facilities The municipality will hold 75% of its programs outdoors The municipality will offer pick-up and drop-off options for equipment loan programs The municipality will offer a “safe routes to school” program The municipality will identify and improve neighbourhood play zones based on existing assets and community support The municipality will create a universal design strategy (inclusive to all abilities) The municipality will provide signage for existing outdoor spaces The municipality will host an annual summit on outdoor play

### 3.2 Post-workshop policy development

Below, we describe the process taken by one community, the Town of Truro, whose policy was adopted by the town council in Fall 2021 (the remaining two communities did not move forward in writing or adopting an outdoor play policy following the workshops). Following the workshops, staff from the Town of Truro and Uplift worked together to develop a community-specific outdoor play policy based on best practices and input from community members, youth, and town staff.

### 3.3 Youth input survey

To gather youth input in Truro, an online survey was distributed through the Truro Youth Council. A more comprehensive consultation was not possible due to the COVID-19 pandemic and staff deployments to COVID-19 related roles. A member of the youth council was a key mobilizer in distributing the survey to students at several local schools. The survey questions were developed by UpLift Partnership staff, recreation staff, and a school administrator. Students (*n* = 194) in grades 9–10 (74%) and 11–12 (26%) responded to the survey to share their thoughts on outdoor play in Truro. When asked how far they lived from downtown Truro, 38% could walk or bike to downtown, 27% lived within a 5–10 min drive, and 35% lived more than a 10-min drive from downtown. Half of the youth (51%) agreed or strongly agreed that Truro has a lot of outdoor spaces for people their age to enjoy being active. Most of youth agreed or strongly agreed that: there is green space within walking distance of where they live (76%) and it is easy to get around Truro on foot (78%). While Truro has a winter equipment loan program, most youth (77%) were not familiar with the program. When youth were asked what gets in the way of youth being active outside in Truro, 48% reported social anxiety, 42% would rather play video games or spend time on social media, 38% felt that Truro does not have outdoor spaces that appeal to youth, 42% reported a lack of transportation to outdoor spaces, and 29% reported their parents’ rules limited time spent outdoors (youth could select multiple responses). Almost half of youth (46%) reported that the main park (Victoria Park, with a playground, trails, pool, and mountain bike park) was their favorite outdoor space in Truro.

### 3.4 Community profile

The Town of Truro has a population of 12,954 and 46,157 in the Truro Census Agglomeration (27). The Parks, Recreation and Culture Department has eight full time staff and 20 maintenance staff, and offers recreation programs, services, and facilities. Truro is described as a very walkable town with an abundance of outdoor play and recreation facilities, including Victoria Park (3,000 acres), ball fields, trails, outdoor play and splash pad, mountain bike park, community gardens, outdoor tennis and basketball courts, soccer fields, skate park, playgrounds, and lawn bowling. The vision statement within the new policy states that “The Town of Truro will be a community where the right to play is available to all children and youth, regardless of age, ability, gender, ethnicity, geographic location, or economic circumstances. The Town of Truro envisions a child friendly environment where play is everywhere, not simply in playgrounds.” The policy’s six goals and policy actions are included in Table 4. Most actions are underway; however, progress has been made for several actions. For example, a garden space was donated to the Boys and Girls Club and the equipment loan program was enhanced through community donations and a Recreation Nova Scotia grant. The updated Town of Truro Active Transportation Strategy (2023) cites the Outdoor Play Policy for Children and Youth as part of its baseline platform to develop the new plan (28). In addition, a development proposal submitted to the Town of Truro in 2021 (after the Policy was approved) included rolling hills to encourage outdoor play and a community outdoor amenity space (29).

**Table 4:** Policy actions and status for the Outdoor Play for Children and Youth Policy in Truro, Nova Scotia.

Policy Goal	Policy Action	Status
Leadership and planning	The Town of Truro will provide leadership and advocacy for outdoor play in the community, during out of school time periods.	
	The Town of Truro will review its policies and bylaws to ensure there are no unintentional barriers to outdoor play.	Complete/ Ongoing
	The Town of Truro will support community groups with training on outdoor play and the development of initiatives such as skateboard parks, community gardens, nature play, loose parts, and play streets.	Ongoing
	The Town of Truro shall host mini gatherings or workshops for organizations that have an interest in outdoor play. These will focus on success stories, learning about outdoor play, and possible partnerships.	
Youth engagement	The Town of Truro will use a variety of methods to engage children and youth in planning and design of outdoor play spaces.	
	The Town of Truro will regularly ask children and youth about play preferences and satisfaction with and awareness of existing spaces and places. E.g., The Town of Truro will focus on a different age group each year.	Ongoing
Land use planning and outdoor play spaces	Land use policies shall encourage formal and informal outdoor play spaces. All improvements to public spaces will be considered as an opportunity to create space for spontaneous or incidental play. Spaces shall incorporate micro elements that encourage participation. Conditions for nature play will be considered a priority in play spaces.	Ongoing
	The Director of Parks, Recreation and Culture will be consulted regarding the need for outdoor play space in any land use planning initiatives.	Ongoing
	The Town of Truro will build on existing play spaces and develop a network of multi functional play spaces, using a system such as neighborhood play space (within 0.5 km) of home; community play space (1 km from home) and destination play space (within Town limits, and easy to get to).	Ongoing
	Vacant and unused lands will be inventoried and assessed for suitability as play spaces (not necessarily a playground).	
	All development agreements shall consider the inclusion of play spaces to satisfy amenity space requirements.	Complete/ Ongoing
	Develop a system to review & renew playgrounds and play spaces to ensure safety, lifecycle maintenance, appropriate signage/directions are consistent with child and youth needs for challenge and risk. Children and youth should participate in the review.	Ongoing
	Play spaces and facilities will be audited using an equity lens and Universal Design Guidelines. This is consistent with new provincial legislation on Accessibility.	
	Play equipment will be made available where children do not have access to playgrounds and play spaces. E.g., The Town of Truro Equipment Loan Programs.	Complete/ Ongoing
Connect community and public spaces	Develop and maintain sidewalks and bike lanes and supporting infrastructure. In a survey of 190 Truro junior and senior high school students, 45% said sidewalks would have the biggest impact on their decision to walk or bike to school. 23% said bike lanes would impact their decisions.	
	Street design will be modified to slow traffic and incorporated into transportation plans.	
Programs to support outdoor play	The Town of Truro will maintain and enhance its equipment loan programs.	Ongoing
	The Town of Truro will develop opportunities for children and youth to learn skills and knowledge that enable participation in outdoor play. These opportunities should complement what is learned at school about outdoor play.	
Communication and awareness	The Town of Truro will plan and implement a communications strategy on Outdoor Play. The key audiences are parents and local decision makers. A key message for parents is the 3-step lifeguard approach developed by Dr. Marianna Brussoni. Observe, check in, and intervene.	
	The Town of Truro will use the existing Fundy Connect database to promote its sport and recreation programs, facilities, greenspaces and parks.	
	The Town of Truro and community agencies shall use special events, information, and pop-up installations to promote outdoor play in public spaces, parks and streets.	

## 4 Discussion

### 4.1 Practical implications

This paper outlines the development process of outdoor play policies in Nova Scotia, including the adoption process for an outdoor play policy for children and youth in Truro, Nova Scotia, Canada. The policy was developed through a unique partnership between the UpLift Partnership, the Town of Truro Parks, Recreation, and Culture Department, including the Director of Recreation and its Municipal Physical Activity Leaders. Workshops, facilitated by UpLift, were attended by various community partners who worked together to brainstorm a vision, goals, and possible policy actions for the Town of Truro. These ideas then informed the development of the formal Outdoor Play for Children and Youth Policy that was adopted by the Town Council in Fall 2021. The adoption of this policy highlights the Town of Truro’s commitment to outdoor play for children and youth and can inform municipal initiatives going forward.

### 4.2 Lessons learned

By completing this project, staff from the Town of Truro and UpLift identified several facilitators to development and implementation of an outdoor play policy in a municipal setting. From this experience with the Town of Truro, municipal commitment, leadership from recreation management and municipal planning staff, and buy-in at the community-level were essential to successful policy development and adoption. Having the policy adopted by Town Council demonstrates political leadership and support for this work across the municipality. In addition to community readiness for a formal policy, it is helpful if the policy aligns with other relevant community policies or strategies, such as Physical Activity or Active Transportation Strategies. For example, The Town of Truro released an updated Physical Activity Strategic Plan in 2020, which included a Strategic Direction for Natural and Built Environments, a fitting connection to the 2021 Outdoor Play for Children and Youth Policy (30). The Town of Truro also has Strategic Priorities to upgrade an outdoor multi-use sport facility and to expand a mountain bike park, further showing the readiness, interest, and investment in outdoor recreation opportunities (28). It was also valuable to involve community partners from different sectors and organizations in the process to ensure the policy is relevant to various community members. The policy actions were also informed by a survey completed by 190 local adolescents that included questions about their interests and needs related to outdoor play. A final facilitator, as identified by the community, was the support of an external facilitator from the UpLift Partnership to develop the outdoor play policy by supporting decision making and the policy writing process.

## 5 Conceptual or methodological constraints

In addition to facilitators to development and implementation, we experienced several barriers. The UpLift Partnership initially supported additional communities to develop outdoor play policies; however, final policies were not developed or adopted as communities lacked readiness and dedicated leadership at the time. We acknowledge the limited generalizability of our findings to other settings or communities with different socio-cultural or environmental factors; however, we do believe our community case study still presents relevant findings and lessons learned for other communities interested in developing outdoor play policies, particularly given the limited literature on this topic. Due to the COVID-19 pandemic, we were unable to conduct in-person youth consultations or in-person community workshops. The pandemic also meant that several community partners (i.e., Public Health, Youth Engagement Coordinators) were deployed from their usual roles to COVID-19 related roles, limiting their participation in the process. The online survey and workshops limited interactions between project partners, and any future work we conduct will involve in-person consultations with youth, such as focus groups or community walk-abouts, and in-person community workshops with more interactive, engaging activities.

## 6 Conclusion

Development and adoption of the Outdoor Play for Children and Youth Policy in Truro has potential to provide more opportunities for children and youth to engage in unstructured play in formal and informal spaces, similar to the actions highlighted in the Play-Friendly Cities Framework and Declaration on the Right to Play (4, 5). The process outlined in this paper can serve as a guide for other communities looking to develop similar outdoor play policies. In the future, more support for adoption and implementation would be a helpful addition to this project for the policy to have the most impact in the community. Future work should continue to evaluate the policy and its impact on activities and initiatives within the Town of Truro, and the development and adoption of polices in other municipalities.

## Data availability statement

The raw data supporting the conclusions of this article will be made available by the authors, without undue reservation.

## Author contributions

HC: Formal analysis, Methodology, Project administration, Resources, Writing – original draft. MA: Conceptualization, Investigation, Project administration, Resources, Writing – review & editing. AS: Project administration, Resources, Writing – review & editing. HM: Project administration, Writing – review & editing. CH: Conceptualization, Funding acquisition, Writing – review & editing. SK: Conceptualization, Funding acquisition, Resources, Writing – review & editing.

## References

[1] United Nations Children’s Fund. Guidance Note: The Child Friendly Cities Initiative. (2022). Available at: https://www.unicef.org/media/133746/file/Child-Friendly_Cities_Initiative_Guidance_Note.pdf

[2] UNICEF. Convention on the rights of the child. (1989). Available at: https://www.unicef.org/child-rights-convention/convention-text#

[3] International Secretariat for Child Friendly Cities. Building child friendly cities: A framework for action. Innocenti publications. Florence, Italy: UNICEF Innocenti Research Centre (2004).

[4] International Play Association Canada. Declaration on the Child’s right to play in Canada. (2021). Available at: https://www.ipacanada.org/declaration/

[5] Canadian Public Health Association. Becoming a play-Friendly City. (2019). 1–7. Available at: https://www.cpha.ca/becoming-play-friendly-city

[6] Caldwell HAT Yusuf J Arthur M Hancock Friesen CL Kirk SFL. Play-friendly communities in Nova Scotia, Canada: a content analysis of physical activity and active transportation strategies. Int J Environ Res Public Health. (2022) 19:2984. doi: 10.3390/ijerph19052984 PMC8910746 35270678

[7] Poitras VJ Gray CE Borghese MM Carson V Chaput J-P Janssen I . Systematic review of the relationships between objectively measured physical activity and health indicators in school-aged children and youth. Appl Physiol Nutr Metab. (2016) 41:S197–239. doi: 10.1139/apnm-2015-0663, PMID: 27306431

[8] Carson V Hunter S Kuzik N Gray CE Poitras VJ Chaput J-P . Systematic review of sedentary behaviour and health indicators in school-aged children and youth: an update. Appl Physiol Nutr Metab. (2016) 41:S240–65. doi: 10.1139/apnm-2015-0630, PMID: 27306432

[9] Gray C Gibbons R Larouche R Sandseter E Bienenstock A Brussoni M . What is the relationship between outdoor time and physical activity, sedentary behaviour, and physical fitness in children? A systematic review. Int J Environ Res Public Health. (2015) 12:6455–74. doi: 10.3390/ijerph120606455, PMID: 26062039 PMC4483711

[10] Lee EY Bains A Hunter S Ament A Brazo-Sayavera J Carson V . Systematic review of the correlates of outdoor play and time among children aged 3-12 years. Int J Behav Nutr Phys Act. (2021) 18:1–46. doi: 10.1186/s12966-021-01097-9, PMID: 33736668 PMC7972019

[11] Pate RR Trilk JL Byun W Wang J. Policies to increase physical activity in children and youth. J Exerc Sci Fit. (2011) 9:1–14. doi: 10.1016/S1728-869X(11)60001-4

[12] Rakhimova N McAslan D Pijawka D. Measuring child-friendly cities: developing and piloting an indicator assessment tool for sustainable neighborhood planning. J Urban. (2022):1–27. doi: 10.1080/17549175.2022.2111589

[13] de Lannoy L Rhodes RE Moore SA Faulkner G Tremblay MS. Regional differences in access to the outdoors and outdoor play of Canadian children and youth during the COVID-19 outbreak. Can J Public Heal. (2020) 111:988–94. doi: 10.17269/s41997-020-00412-4 PMC7556599 33057923

[14] Caldwell HAT Faulkner G Tremblay MS Rhodes RE de Lannoy L Kirk SFL . Regional differences in movement behaviours of children and youth during the second wave of the COVID-19 pandemic in Canada: follow-up from a national study. Can J Public Heal. (2022) 113:535–46. doi: 10.17269/s41997-022-00644-6, PMID: 35507303 PMC9066998

[15] ParticipACTION. Position statement on active outdoor play. Toronto: (2015). 10.3390/ijerph120606475 PMC4483712 26062040

[16] Government of Nova Scotia. Let’s get moving Nova Scotia: an action plan for increasing physical activity in Nova Scotia. (2018). Available at: https://novascotia.ca/letsgetmoving/docs/letsgetmoving-en.pdf

[17] Province of Nova Scotia. Minimum planning requirements regulations - municipal government act. (2019). Available at: https://novascotia.ca/just/regulations/regs/mgaminimum.htm (Accessed November 1, 2023).

[18] Goins KV Schneider KL Brownson R Carnoske C Evenson KR Eyler A . Municipal officials’ perceived barriers to consideration of physical activity in community design decision making. J Public Health Manag Pract. (2013) 19:S65–73. doi: 10.1097/PHH.0b013e318284970e PMC4928376 23529058

[19] Palmerston North City Council. Play Palmy, Play. (2021). Available at: https://www.pncc.govt.nz/files/assets/public/documents/council/policies/play-policy-2021.pdf

[20] YYC Plays. Calgary’s play charter. Calgary: (2017).

[21] Aberdeen City. Aberdeen City play policy and strategy. (2018). Available at: https://www.aberdeencity.gov.uk/sites/default/files/2018-06/AberdeenCityPlayPolicyandStrategy2018.pdf

[22] Smith ML Levkoff SE Ory MG. Community case study article type: criteria for submission and peer review. Front Public Heal. (2016) 4:175843. doi: 10.3389/fpubh.2016.00056, PMID: 27148510 PMC4830820

[23] Kontak JCH Caldwell HAT Quann EC Hancock Friesen CL Machat S Barkhouse K . Health promoting schools in Nova Scotia: past, present, and future. PHE Canada J. (2023) 1:88

[24] Joint Consortium for School Health. What is comprehensive school health? (2016). Available at: https://www.jcsh-cces.ca/en/concepts/comprehensive-school-health/

[25] World Health Organization, UNESCO. Making every school a health promoting school -- global standards and indicators for health-promoting schools and systems. (2021). Available at: https://www.who.int/publications/i/item/9789240025059

[26] Bassett-Gunter R Yessis J Manske S Stockton L. Healthy school communities concept paper. Ottawa: Physical & Health Education Canada (2012).

[27] Canada Statistics. Profile table, census profile, 2021 census of population - Nova Scotia. Available at: https://www12.statcan.gc.ca/census-recensement/2021/dp-pd/prof/details/page.cfm?Lang=E&SearchText=NovaScotia&DGUIDlist=2021A000212&GENDERlist=1,2,3&STATISTIClist=1&HEADERlist=0

[28] Town of Truro. Strategic initiatives. (2023). Available at: https://www.truro.ca/strategic-initiatives/

[29] Town of Truro. Development applications [555–573 Prince Street]. (2023). Available at: https://townoftruro.maps.arcgis.com/apps/Shortlist/index.html?appid=6ec955d1bdc94ad991ca5f3ae5b7d5e8

[30] Town of Truro. Physical activity strategic plan 2020–2025. Truro: (2020) Available at: https://www.truro.ca/government/policies.

